# Ipilimumab-induced thrombotic thrombocytopenic purpura (TTP)

**DOI:** 10.1186/s40425-017-0224-7

**Published:** 2017-03-21

**Authors:** Jeanelle King, Javier de la Cruz, Jose Lutzky

**Affiliations:** 1 Mount Sinai Comprehensive Cancer Center, Division of Hematology/Oncology, 4306 Alton Road, Miami Beach, FL 33140 USA; 2 Mount Sinai Medical Center, Department of Internal Medicine, 4300 Alton Road, Miami Beach, FL 33140 USA

## Abstract

**Background:**

CTLA-4 (Cytotoxic T-lymphocyte-associated protein 4) was the first immune checkpoint receptor clinically targeted for use in cancer treatment. It is expressed exclusively on T-cells where its primary role is to regulate the amplitude of the early stages of T-cell activation.1 Ipilimumab, a CTLA-4 blocking antibody, has been widely used for the treatment of patients with high risk and metastatic melanoma. Given its mechanism of action and consequent immune activation, the side effect profile of this drug greatly differs from that of standard cytotoxic chemotherapy. Adverse events are from the most part immune-mediated, ranging from the more common, such as rash and fatigue, to the less common, such as immune endocrinopathy and colitis.

**Case presentation:**

We describe a case of immune-mediated thrombotic thrombocytopenic purpura (TTP) in a 68 year-old woman with high risk, stage III melanoma occurring after 3 cycles of adjuvant treatment with ipilimumab as part of a clinical trial.

**Conclusion:**

The range of immune-mediated adverse events during treatment with ipilimumab is wide and varied and clinicians should have a high degree of suspicion when managing these patients.

## Background

CTLA-4 (Cytotoxic T-lymphocyte-associated protein 4) was the first immune checkpoint receptor clinically targeted for use in cancer treatment. It is expressed exclusively on T-cells where its primary role is to regulate the amplitude of the early stages of T-cell activation [1]. Ipilimumab, a CTLA-4 blocking antibody, has been widely used for the treatment of patients with high risk and metastatic melanoma. Given its mechanism of action and consequent immune activation, the side effect profile of this drug greatly differs from that of standard cytotoxic chemotherapy. Adverse events are from the most part immune-mediated, ranging from the more common, such as rash and fatigue, to the less common, such as immune endocrinopathy and colitis [2]. Here, we describe a case of immune-mediated thrombotic thrombocytopenic purpura (TTP) in a 68 year-old woman with high risk, stage III melanoma occurring after 3 cycles of adjuvant treatment with ipilimumab as part of a clinical trial.

## Case presentation

A 68 year old woman with stage III (pT3,N2,M0) ulcerated spindle cell melanoma arising from the anterior aspect of the right inferior turbinate mucosa was referred to our melanoma clinic following definitive surgery. She agreed and signed informed consent for participation in an adjuvant study in which she was randomized to receive ipilimumab, an anti-CTLA-4 antibody, at 10 mg/kg IV every 3 weeks for 4 cycles followed by maintenance therapy.

After the first cycle on 6/29/2015, she developed a grade 1 pruritic rash and grade 1 ALT and AST elevation. She received cycle 2 on July 20, 2015. Prior to receiving cycle 3, transaminases increased further to grade 2. As a result, cycle 3 was held and the patient was started on prednisone 40 mg daily, and then tapered over 4 weeks to 5 mg prednisone daily. The transaminase elevation decreased from grade 2 to grade 1 and cycle 3 was given on 8/24/15. During that time, she was found to have an otitis externa and was prescribed topical ciprofloxacin + dexamethasone drops from 8/24/2015 to 9/10/2015, followed by oral clindamycin 300 mg TID from 9/1/2015 to 9/11/2015. When seen on 9/9/2015, she had grade 1 transaminase elevation, mild hyperglycemia, normal renal function and a very mild microcytic anemia. Platelet count and leukocyte count were normal.

**Fig. 1:**
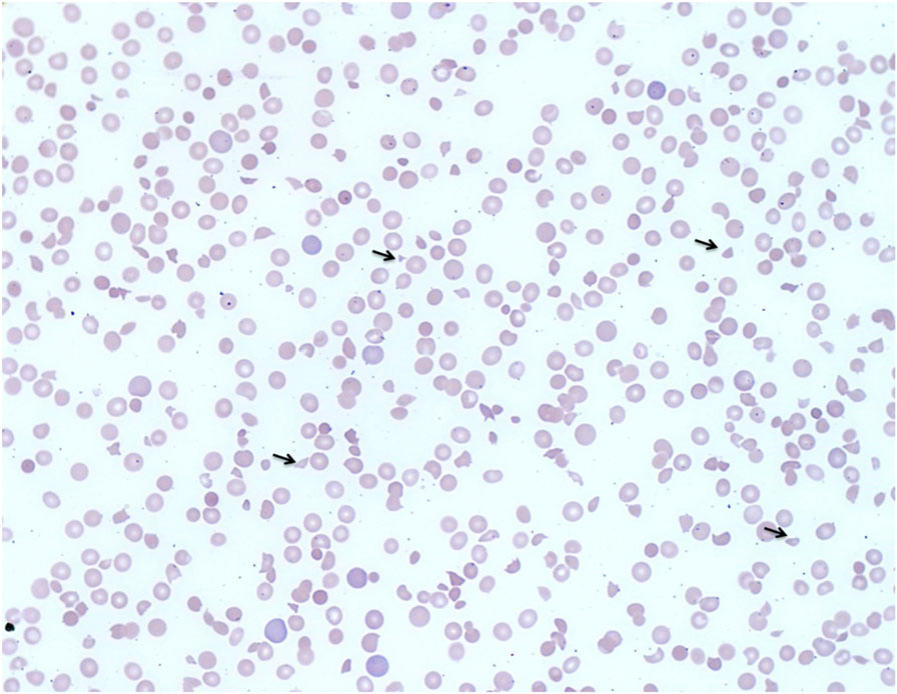
Peripheral blood smear demonstrating extensive presence of schistocytes

On September 12, 2015, the patient developed profound fatigue, decreased PO intake, nausea, vomiting, and two episodes of diarrheal stools. She presented to clinic on September 14, 2015. On the morning of her evaluation, she complained of subjective fevers, altered mental status, and worsening fatigue. Physical examination was remarkable for a petechial rash over the chest and bilateral upper extremities. Laboratory evaluation demonstrated a severe microcytic anemia and thrombocytopenia, with a hemoglobin of 6.8 g.dL and a platelet count of 7,000/uL. There was mild leukocytosis with a WBC of 12,700/uL. Examination of the peripheral blood smear (Fig. 1) showed marked anisocytosis, severe decrease in platelets and the presence of abundant schistocytes. Reticulocyte count was 5.41% (ULN 2.1%), serum LDH was 7,329 (ULN 618 U/L) and serum haptoglobin was < 7.8 mg/dL (LLN 30 mg/dL). Acute renal failure was characterized by oliguria, serum creatinine of 2.8 mg/dL and BUN 98 mg/dL. Serum transaminases were mildly elevated. Serum bilirubin was 2.1 mg/dL (ULN 1.3 mg/dL). Sedimentation rate was 75 mm/h (ULN 30 mm/h) and serum C-reactive protein was 18.7 mg/L (ULN 3 mg/L). Blood cultures and disseminated intravascular coagulation panel were negative. ADAMTS13 activity and inhibitor level were collected upon admission to the hospital.

**Fig. 2:**
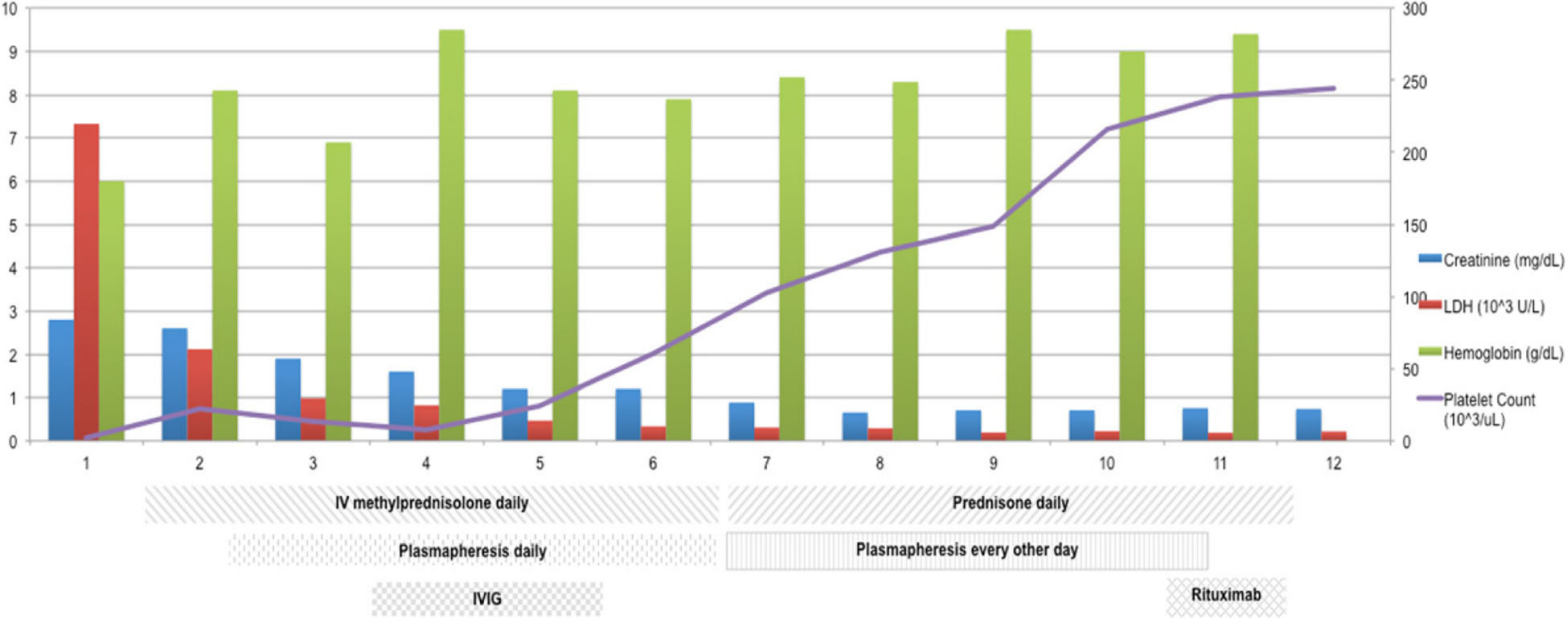
*Vertical axis:* values of serum creatinine, lactate dehydrogenase (LDH), hemoglobin, and platelet count during hospitalization in relation to treatment with plasmapheresis, systemic corticosteroids, intravenous immunoglobulin (IVIG) and rituximab therapy. *Horizontal axis:* time in days

The patient was admitted to the intensive care unit with a diagnosis of TTP. Solu-Medrol 1000 mg IV was given for potential immune-mediated ipilimumab toxicity. During hospitalization, the patient received the following interventions: 5 courses of plasmapheresis with plasma exchange (9/16, 9/18, 9/19, 9/21, and 9/23), transfusion of 4 units of packed red blood cells, one unit of platelets, IV gammaglobulin 2 g over two days (9/17 and 9/18), and rituximab 375 mg/m^2^ (9/24/15) (Fig. 2).

She improved clinically over the next several days with reversal of renal failure, increased hemoglobin and haptoglobin, normalization of LDH, and no recurrence of mental status changes. By September 23, 2015, LDH, haptoglobin, renal function, and platelet count were normalized. She was given rituximab on 9/24/15 and discharged home. Results of ADAMTS13, available on day 7, revealed that ADAMTS13 activity was <3% (normal: 68-163% activity); ADAMTS13 inhibitor was 3.7H (normal: <0.4 BEU).

Follow-up as an outpatient has shown no evidence of TTP recurrence to the date of this report. She was permanently taken off ipilimumab and remains under close monitoring for her high-risk melanoma.

## Discussion

TTP is a primary thrombotic microangiopathy manifested by consumptive thrombocytopenia, microangiopathic hemolytic anemia (MAHA), and thrombosis. The underlying pathophysiology of TTP is inhibition/inactivation of A Disintegrin And Metalloprotease with ThromboSpondin type 1 repeats, 13 (ADAMTS13), a metalloprotease responsible for cleaving large multimers of von Willebrand factor (vWF) into smaller units. The increase in circulating large multimers of vWF promotes platelet adhesion to areas of endothelial injury, particularly at arteriole-capillary junctions. As a consequence, passing red blood cells are subjected to shear stress leading to rupture of red blood cells within blood vessels, producing hemolytic anemia and schistocyte formation. Reduced blood flow due to thrombosis and cellular injury, results in end-organ damage.

Many patients also present with neurological symptoms, renal involvement, and fever [3, 4]. The classic pentad of microangiopathic hemolytic anemia, thrombocytopenia, neurologic symptoms, renal failure, and fever, albeit uncommon, has been traditionally considered to be diagnostic of the disease.

While most cases of TTP are caused by ADAMTS13 autoantibodies, secondary TTP can also be caused by drugs, bone marrow transplantation, and HIV infection. TTP may occur due to a congenital deficiency of ADAMTS13 (Upshaw-Shulman syndrome) in about 1% of patients.

Current therapy is based on supportive care and plasmapheresis with plasma exchange to reduce circulating antibodies against ADAMTS13 and to replenish blood levels of the enzyme. Monoclonal therapy with the anti-CD20 antibody rituximab has recently shown efficacy [3–11].

Here, we describe the first report, to our knowledge, of TTP associated with the use of ipilimumab. Although it is possible that this patient could have had secondary TTP from oral clindamycin therapy, a literature search failed to demonstrate clindamycin-induced TTP. There have been 2 cases reported of clarithromycin-induced TTP [12]. Ciprofloxacin has been associated with TTP but our patient was treated with topical ciprofloxacin drops only [13].

Although rare, there have been reports of isolated hemolytic anemia and thrombocytopenia related to Ipilimumab therapy [14]. Given the wide variety of immune manifestations reported with the use of ipilimumab and the autoimmune underlying nature of TTP, TTP should be added to the list of possible immune-related adverse events of ipilimumab and considered in the differential diagnosis of ipilimumab-associated thrombocytopenia.

## Conclusion

With the advent of immunotherapy as a dominant modality in cancer treatment, it is essential for clinicians to be able to quickly identify and treat the common associated and rare immune related toxicities. This requires a high degree of clinical suspicion when addressing patient reported signs and symptoms.

## Abbreviations

ADAMST13: A Disintegrin And Metalloprotease with ThromboSpondin type 1 repeats, 13
BEU: Bethesda units
CTLA-4: Cytotoxic T-lymphocyte-associated protein 4
MAHA: Microangiopathic hemolytic anemia
TTP: Thrombotic thrombocytopenic purpura
ULN: Upper limit of normal

## References

[1] Pardoll D (2015). Cancer and the immune system: basic concepts and targets for intervention. Semin Oncol.

[2] Hodi FS, O’Day SJ, McDermott DF, Weber RW, Sosman JA, Haanen JB (2010). Improved survival with ipilimumab in patients with metastatic melanoma. N Engl J Med.

[3] Moake JL (2002). Thrombotic microangiopathies. N. Engl. J. Med.

[4] George JN, Nester CM (2014). Syndromes of thrombotic microangiopathy. N Engl J Med.

[5] Knöbl P (2014). Inherited and acquired thrombotic thrombocytopenic purpura (TTP) in adults. Semin Thromb Hemost.

[6] Shenkman B, Einav Y (2014). Thrombotic thrombocytopenic purpura and other thrombotic microangiopathic hemolytic anemias: diagnosis and classification. Autoimmun Rev.

[7] Sarode R, Bandarenko N, Brecher ME, Kiss JE, Marques MB, Szczepiorkowski ZM (2014). Thrombotic thrombocytopenic purpura: 2012 American Society for Apheresis (ASFA) consensus conference on classification, diagnosis, management, and future research. J Clin Apher.

[8] Jian C, Xiao J, Gong L, Skipwith CG, Jin SY, Kwaan HC (2012). Gain-of-function ADAMTS13 variants that are resistant to autoantibodies against ADAMTS13 in patients with acquired thrombotic thrombocytopenic purpura. Blood.

[9] Fujikawa K, Suzuki H, McMullen B, Chung D (2001). Purification of human von Willebrand factor-cleaving protease and its identification as a new member of the metalloproteinase family. Blood.

[10] Levy GG, Nichols WC, Lian EC (2001). Mutations in a member of the ADAMTS gene family cause thrombotic thrombocytopenic purpura. Nature.

[11] Rock GA, Shumak KH, Buskard NA, Blanchette VS, Kelton JG, Nair RC (1991). Comparison of plasma exchange with plasma infusion in the treatment of thrombotic thrombocytopenic purpura. Canadian Apheresis Study Group. N Engl J Med.

[12] Hashmi HR, Diaz-Fuentes G, Jadhav P, Khaja M (2015). Ciprofloxacin-Induced Thrombotic Thrombocytopenic Purpura: A Case of Successful Treatment and Review of the Literature. Case Rep Crit Care.

[13] Alexopoulou A, Dourakis SP (2002). Thrombotic thrombocytopenic purpura in a patient treated with clarithromycin. European Journal of Haematology.

[14] Weber J (2007). Review: anti-CTLA-4 antibody ipilimumab: case studies of clinical response and immune-related adverse events. Oncologist.

